# Tuberculosis

**Published:** 1904-09-17

**Authors:** 


### Progress in Medicine and Surgery.

### TUBERCULOSIS.

*(Concluded from page 417.)*

**Sanatorium Treatment.**—As regards the class of case suitable for sanatorium treatment Cattle [6] agrees with Burton Fanning that the disease should be limited to one lobe, without clinical signs of cavitation. He would also admit a good many cases of pleurisy, especially if followed by signs of general ill-health. He thinks some chronic cases without hæmoptysis in middle-aged persons do well, even though cavities are present. Bardswell and Chapman consider that in order to obtain the best results the patients should be young, in an early stage of the disease, and able to adopt a suitable employment after treatment. This would in most cases among working men mean that they should be unmarried; and though this view is supported by Fowler, Newsholme from the public point of view thinks that the wage-earners of the family are the most important and should be carefully looked after. As regards the length of stay in sanatoria the writer of a leader in the *British Medical Journal* [7] considers that three months is far too short a time, while Lawson [15] points out that in 1903 the average stay of those discharged from the Banchory sanatorium was seven and a half months, and considers that this, among other factors, accounts for the much better results obtained in private paying institutions than in those designed for the poor. The economic value is treated of by Bardswell and Chapman,[8] who state that, after four years, of 9 cases who had been discharged completely restored to their working capacity, 3 had died, and 4 (possibly 6) were in good health; of 7 who would only perform light work, only 1 was alive. Williams [9] points out, however, that better results had been obtained by Pollock, who had kept a much larger number of patients under observation; while among paying patients Lawson finds that 36·7 per cent. of those treated during the last three years are able to carry on their ordinary vocations. Parkes [10] thinks that if it were possible to select the cases, sanatorium treatment would be economical, as those who would otherwise be incapacitated by disease and come on the rates would be cured and able to earn their living. From a public health point of view, all authors consider even a short stay at a sanatorium advantageous, in teaching the patient how to treat himself and to prevent the infection of others. Parkes quotes the good results obtained in this way at Brighton after one month's education. He also points out that by withdrawing foci of infection some reduction in the prevalence of consumption should occur. From a practical point of view he advocates the conversion of the small-pox convalescent hospitals at Darenth into sanatoria, which, on a basis of three months' treatment, would provide for more than a quarter of the consumptive persons in London. He does not consider that it is probable that they will be required for their original purpose, as an epidemic in London of any magnitude seems at present unlikely. Coupled with this he would make cases of consumption notifiable, if in the opinion of the medical attendant they were suitable for sanatorium treatment or likely to be a source of danger to others. Sykes [11] points out that the French method of classifying cases into "open" and "latent" simplifies matters considerably. He advises that sanatory authorities should examine all specimens of sputum sent them, free of charge, with statutory notification on positive results. He thinks that sanatorium treatment should be given to early cases, and approves of the scheme for the conversion of the Darenth hospitals. "Confirmed" cases should be treated in hospitals, and for "advanced" cases special provision should be made in workhouse infirmaries. Douglas Powell,[12] Ewart,[13] and Cattle recommend taking cases into general hopitals as a preliminary to sanatorium treatment, for the purpose of estimating their suitability for the latter and providing any special treatment necessary. Meanwhile Klebs [14] recommends the establishment of dispensaries with a staff of visiting nurses to select cases for sanatoria, hospitals, etc., to remove children from tuberculous parents, to treat out-patients, and generally give instruction in the practical hygiene of tuberculosis. As regards the line of treatment to be adopted in sanatorium cases, the same writer approves a full and varied diet, but not a "stuffing" process, hydrotherapy, and carefully selected occupation rather than absolute rest or inaction. Lawson [15] strongly advocates hill-climbing, in a small way only till the patient can walk six miles on the flat without rise of temperature. Later, 1,000 to 1,500 feet may be climbed with advantage at least three times a week. The chronic fibroid cases, however, are not benefited by it. He does not consider heavy dieting necessary, but insists on great variety and a small amount of every kind of food. The diet should be scientifically based on the observed metabolism of the patient, and the proper amount calculated in calories, when weighed meals can be given. He has found great advantage from the careful supervision of the teeth, and considers high-frequency currents valuable in the cases of atonic dilatation of the stomach. The *x*-rays he finds useful in tuberculous disease of the glands and lupus. A curious point resulting sometimes from sanatorium treatment, noted by Lyon [16] and Pitt,[17] is a certain mental and moral degeneration which unfits the patient for subsequent work in the world. While all authorities seem agreed on the value of the treatment in selected cases, it is generally admitted that the tendency to relapse is great. Bardswell and Chapman found satisfactory occupations for discharged patients were telephone company's labourer, steward of a working-men's club, tram conductor, rate collector, workhouse infirmary attendant, van driver, and hall porter. Fowler [18] considered market gardening a good employment, and said that for two years after arrest, walking should be the most violent form of exercise allowed. Sykes, Fowler, Williams, Douglas, Powell, and Pitt are all advocates of the establishment of "colonies" for patients discharged from sanatoria. Lawson does not believe that residence abroad offers any advantages to those in whom tuberculosis is still active, but finds that quiescent cases, where the lung is already severely damaged, are the better for escaping the English winter. He also condemns the practice of sending patients abroad before they have been properly instructed in sanatoria at home.

Pathogenesis.—Congenital tuberculosis is generally considered to be usually inherited from the mother, and Warthen and Cowie [19] have recently published a case of this kind. The woman died in the seventh month of pregnancy, and besides other lesions the placenta, decidua and the fœtus itself were found to be infected. It appears to them un-uncertain whether an intact placenta will allow the passage of bacilli, but as the placenta normally degenerates before birth it is possible that the atrophic or necrotic patches are the points through which fœtal infection occurs. It further appears that the fœtal tissues are relatively immune, so that the disease may remain latent for some time after birth. The frequency of this mode of infection has not been determined but is thought by the writers to be more common than is usually supposed. The theory of Koch as to the differences between human and bovine tuberculosis has been examined by a committee of experts whose report is published by the German Imperial Health Office.[20] While holding that Koch was, generally speaking, right in asserting the essential dissimilarity between the two strains, they found in one instance that typical "perlsucht" resulted in a calf from the intravenous injection of bacilli from a case of general tuberculosis in a child, and so they conclude that no relaxation should yet be permitted in the precautions taken to prevent milk infection. Römer [21] has shown that human tubercle bacilli vary much in their effects on cattle, and in his immunisation experiments he used one particular strain of human origin which alone was found to give satisfactory results. The German committee above mentioned have also shown that avian tubercle is transmissible to mammals, but not *vice versâ*. Ravenel [22] observes that, apart from the transmission of human tubercle to animals, which has been already proved to occur, in four cases he has seen the accidental inoculation of humans with bovine bacilli, and quotes many cases recorded by others. With regard to infection by food, he thinks that this occurs in a certain number of cases, mostly in infants, though accurate proof is difficult. In England primary intestinal tuberculosis occurs in rather more than 25 per cent. of the cases. Heller, though giving only 1·43 per cent. of primary intestinal cases, found the principal lesion to lie in the abdomen in 37·8 per cent. Hueppe gives the average in Germany as 25 per cent. to 30 per cent. These figures are, of course, obtained from post-mortem examinations.

[6] Med. Rec, March 12, 1904, p. 409. [7] Brit. Med. Jour., January 9, 1904. [8] Ibid., April 30, 1904, p. 1016. [9] Ibid, [10] Pract., April 1904, p. 601. [11] Brit. Med. Jour., February 13. 1904, p. 368. [12] Ibid., April 30, 1904, p. 1017. [13] Ibid. [14] Med. Rec., February 20, 1904, p. 300. [15] Lancet, May 21, 1904, p. 1420. [16] Brit. Med. Jour., April 30, 1904, p. 1017. [17] Ibid. [18] Ibid. [19] Med. Rec., April 23, 1904, p. 657. [20] Brit. Med. Jour., April 9, 1904, p. 847. [21] Ibid. [22] Med. Rec., April 2, 1904, p. 540.

